# Supplementary material for: Optical force-induced nonlinearity and self-guiding of light in human red blood cell suspensions
Source: Light Sci Appl. 2019 Mar 13;8:31. doi: 10.1038/s41377-019-0142-1 (PMC6414597; doi:10.1038/s41377-019-0142-1)
Supplement: Supplementary file 1 — Supplementary Material [file 41377_2019_142_MOESM1_ESM.pdf]

# Optical force-induced nonlinearity and self-guiding of light in human red blood cell suspensions

Rekha Gautam[1,2*], Yinxiao Xiang[1,3*], Josh Lamstein[1*], Yi Liang[1,4], Anna Bezryadina[1,5], Guo Liang[1], Tobias Hansson[6,7], Benjamin Wetzel[6,8], Daryl Preece[9], Adam White[1], Matthew Silverman[10], Susan Kazarian[10], Jingjun Xu[3], Roberto Morandotti[6,11,12], and Zhigang Chen[1,3]

[1]*Department of Physics and Astronomy, San Francisco State University, San Francisco, CA 94132, USA*

[2]*Department of Biomedical Engineering, Vanderbilt University, Nashville, TN 37240, USA*

[3]*MOE Key Lab of Weak-Light Nonlinear Photonics, TEDA Applied Physics Institute and School of Physics, Nankai University, Tianjin 300457, China*

[4]*Guangxi Key Lab for Relativistic Astrophysics, Guangxi Colleges and Universities Key Lab of Novel Energy Materials and Related Technology, School of Physical Science and Technology, Guangxi University, Nanning, Guangxi 530004, China*

[5]*Department of Physics and Astronomy, California State University Northridge, Northridge, CA 91330, USA*

[6]*Institut National de la Recherche Scientifique, Université du Québec, Varennes, Québec J3X 1S2, Canada*

[7]*Department of Physics, Chemistry and Biology, Linköping University, Linköping SE-581 83, Sweden*

[8]*School of Mathematical and Physical Sciences, University of Sussex, Sussex House, Falmer, Brighton BN1 9RH, UK*

[9]*Department of Biomedical Engineering, University of California Irvine, Irvine, CA, USA*

[10]*Clinical Laboratory Science Program, San Francisco State University, San Francisco, CA 94132, USA*

[11]*Institute of Fundamental and Frontier Sciences, University of Electronic Science and Tech. of China, Chengdu 610054, China*

[12]*ITMO University, Saint Petersburg 197101, Russia*

*These authors made equal contribution.

Corresponding author: zhigang@sfsu.edu, yinxiaoermao@nankai.edu.cn

# Supplementary Information

## 1. Animation and videos:

Illustration of optical self-trapping by means of red blood cells (RBCs) and the consequent realization of an induced waveguide is depicted in supplementary Fig. S1. The corresponding animation (Movie 1) shows the dynamic formation of a biological waveguide in the RBC suspension. The other short videos (Movie 2-10) present RBC movement under the action of the optical forces from the trapping laser beam (532 nm and 960 nm) in different osmotic solutions recorded using either a 100x oil immersion objective (NA=1.3) or a 40x oil immersion objective (NA=0.65), which show that the drift dynamics of RBCs towards the trapping beam (position marked by a circle) is dependent on the NA, the laser beam power, as well as osmotic conditions.

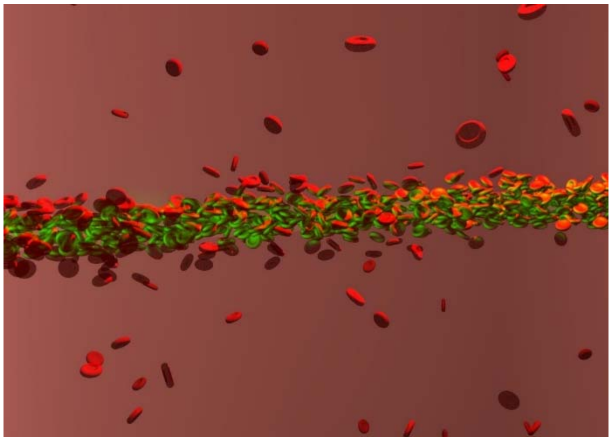

Supplementary Fig. S1: Animation of RBCs moving inward and forward due to the action of optical forces, forming an effective waveguide of light. See supplementary video.

## 2. Absorption spectra of human RBC suspensions:

RBCs in buffer media have a strong Soret band at 416 nm and weak absorption bands at 543 nm and 578 nm (Fig. S2). The measured data for three different buffers were normalized via dividing the spectra by their norms. RBCs have relatively low absorption at 532 nm, the wavelength used in our nonlinear self-trapping experiment. A slight variation in absorption under different buffers can be seen in the zoom-in inset of Fig. S2: the absorbance increases in RBC suspensions when ranging from hypotonic, then isotonic and finally hypertonic buffer conditions. Meanwhile, due to the change of RBC size/shape, the Hb concentration increases within the cell from hypotonic to hypertonic via isotonic buffer conditions, which leads to an increase in the effective index of refraction[22,42].

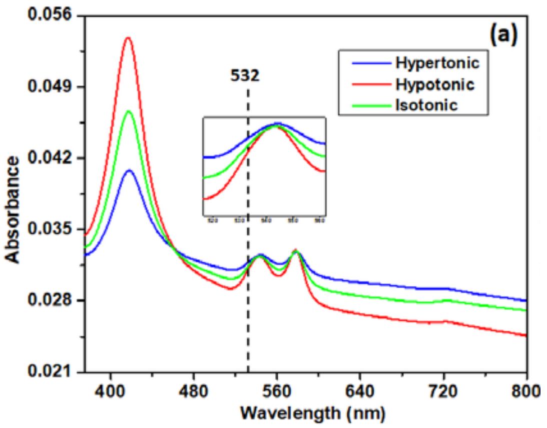


Supplementary Fig. S2: Absorption spectra of RBC suspensions in three different osmotic solutions. The vertical dashed line shows the laser wavelength of 532 nm used in the experiments.

## 3. Transmission measurements in human RBC suspensions:

We performed a series of experiments to measure the normalized transmission of light through the RBC suspensions as a function of input power, under different osmotic conditions. In addition to the summarized results depicted in Fig. 2a, we present here separate plots for each condition where the nonlinear trend is also clearly visible (Fig. S3) – we recall the significant difference in transmission in the three buffers. The normalized transmission (percentage of output vs input power) shows a clear nonlinear upward trend for each case. This could be fitted with, for example, the polynomial order 4, which would not be possible if the propagation dynamics were linear. The value of the adjusted $R^2$ (quantifying the fit accuracy) are 0.932, 0.954, 0.979 in three different isotonic, hypotonic and hypertonic suspensions, respectively.

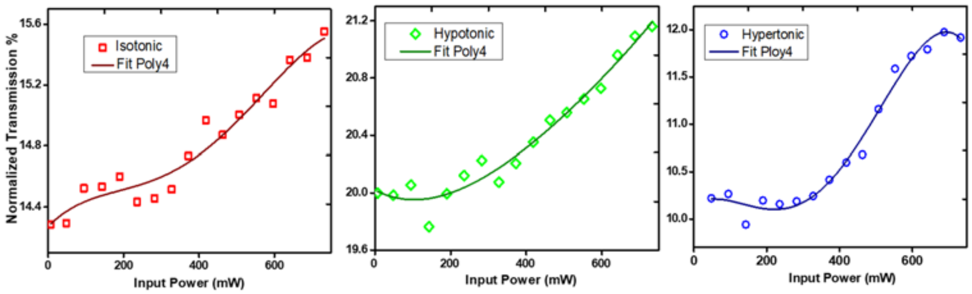


Supplementary Fig. S3: Measured normalized transmission of light through the RBC suspensions in three different osmotic solutions.

**4. Viability assessment of RBCs:**

In this study, a light beam was focused using a normal lens (f = 125 mm) which generates a much lower photon density compared to the high numerical aperture objective (NA ≥ 1.3) used for our optical tweezers setting. Even with an incident power as high as 700 mW, we estimated that the power density (<1 mW$\mu m^{-2}$) is still below the threshold for radiation damage [27]. In the nonlinear propagation experiment, the measurements were performed in 4 mL volume suspensions, which further minimizes the local heating and photodamage effects.

In particular, to check for possible photodamage, the suspended RBCs in all three used buffers were spun down after illumination with the 532 nm laser for several minutes at the power of about 450 mW (higher than the value used for the nonlinear self-trapping experiments of Fig. 1 in different buffer solutions). No pink coloration in the supernatant solution was observed, thus indicating that there is no hemolysis due to the laser illumination. This was verified by recording absorption spectra of the supernatant obtained from the suspensions with and without laser exposure, where no absorption was observed. In addition, to examine possible damage of individual cells due to laser illumination, a high concentration of RBCs (suspended in the hypotonic buffer) were casted on a thin glass slide chamber made of coverslips and exposed to the focused laser beam at 450 mW. White light images were recorded before and after exposure using a 100x oil immersion microscope objective and a CCD camera (Fig. S4). The experiment was repeated three times, and no significant changes in cell shape or damage of the RBCs was observed under the microscope.

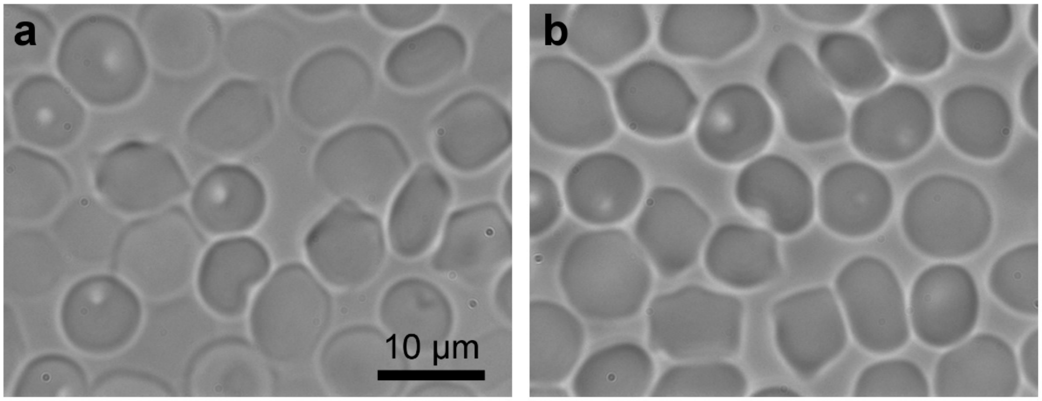


Supplementary Fig. S4: White-light images of RBCs (a) before and (b) after illumination with a 532 nm laser beam at 450 mW power. Images were recorded using a 100x oil immersion microscope objective. No significant changes in shape or damage of the RBCs were observed.

**5. Theoretical model:**

To numerically simulate the nonlinear beam propagation through different suspensions of RBCs, we employed an extension of our non-local theoretical model (previously described in Ref. 21). It is a diffractive nonlinear Schrödinger-type equation:

$$i\frac{\partial\varphi}{\partial z}+\frac{1}{2k_0n_b}\nabla_\perp^2\varphi+k_0V\left(n_p-n_b\right)\rho(\boldsymbol{r})\varphi+i\frac{\sigma}{2}\rho(\boldsymbol{r})\varphi=0, \qquad (1)$$

where $\varphi$ is the electric field envelope, $k_0 = 2\pi/\lambda_0$ denotes the vacuum wavenumber, and $\sigma$ is the scattering cross-section for the absorption losses. Meanwhile, $V$ represents the volume of an individual particle and $n_p$ its refractive index, $n_b$ stands for the refractive index of the background medium, and $\rho$ denotes the time and intensity-dependent particle concentration. The evolution of the latter is determined by coupling Eq. (1) to a diffusion-advection equation:

$$\frac{\partial\rho}{\partial t}+\nabla\cdot(\rho\boldsymbol{v}(\boldsymbol{r})-D\nabla\rho)=0, \qquad (2)$$

where $D$ is the diffusion coefficient, $t$ is time, $\boldsymbol{v} = \mu\boldsymbol{F}(|\varphi|^2)$ is a velocity field determined by the optical forces and $\mu$ is the particle mobility. The intensity dependent optical forces acting on the particles are modeled as $\boldsymbol{F}(I = |\varphi|^2) = \alpha\nabla I + \beta I\hat{z}$, which includes contributions from both an optical gradient force with polarizability coefficient $\alpha$ and a forward-scattering force with coefficient $\beta$ along the longitudinal direction $\hat{z}$. In our simulations, we used $\alpha = 1.2\cdot10^{-27}$ $m^2$.s and $\beta = 1.2\cdot10^{-18}$ m.s , which results in a proportionality of $\beta\ /\alpha$ consistent (i.e., in the same order of magnitude) with Rayleigh predictions. The variation in osmotic conditions for the different RBC suspensions is taken into account within the model by changing the particle volume and refractive index, and also by rescaling the magnitude of the optical forces with the assumption of a similar proportionality dependence on size and refractive index as for dielectric spheres in the Rayleigh scattering approximation[51].

To numerically solve Eq. (1), we used a (2+1)D split-step Fourier algorithm that also includes additional scattering effects to model the random fluctuations of the refractive index. To obtain a self-consistent solution, we repeatedly propagate the field through the entire medium and calculate the particle distribution after a short time-step for the corresponding optical force. The new particle distribution is then used in the next iteration to propagate the field again, and the process is repeated until no significant modification of either the field or the particle distribution is observed.

Supplementary Movie 1: Dynamic formation of a biological waveguide in the RBC suspension;
Supplementary Movies 2-4: RBC movement driven by optical forces of the 532 nm trapping beam (100 mW, NA=1.3) in isotonic, hypotonic and hypertonic solutions.
Supplementary Movies 5-7: RBC movement driven by optical forces of the 960 nm trapping beam (100 mW, NA=1.3) in isotonic, hypotonic and hypertonic solutions, corresponding to Fig. 3(a), Fig. 3(b) and Fig. 3(c), respectively.
Supplementary Movie 8: RBC movement driven by optical forces of the 960 nm trapping beam (100 mW, NA=0.65) in hypertonic solution for comparison with Movie 7 taken at same conditions except for NA=1.3.
Supplementary Movies 9-10: RBC movement driven by optical forces of the 532 nm trapping beam (50 mW, NA=0.65, and 1.3) in hypertonic solutions, showing “self-cleaning” of cells in the observing plane due to the strong scattering force at 532 nm.
